# Molecular simulations of lipid membrane partitioning and translocation by bacterial quorum sensing modulators

**DOI:** 10.1371/journal.pone.0246187

**Published:** 2021-02-09

**Authors:** Tianyi Jin, Samarthaben J. Patel, Reid C. Van Lehn

**Affiliations:** Department of Chemical and Biological Engineering, University of Wisconsin – Madison, Madison, Wisconsin, United States of America; University of Helsinki, FINLAND

## Abstract

Quorum sensing (QS) is a bacterial communication process mediated by both native and non-native small-molecule quorum sensing modulators (QSMs), many of which have been synthesized to disrupt QS pathways. While structure-activity relationships have been developed to relate QSM structure to the activation or inhibition of QS receptors, less is known about the transport mechanisms that enable QSMs to cross the lipid membrane and access intracellular receptors. In this study, we used atomistic MD simulations and an implicit solvent model, called COSMOmic, to analyze the partitioning and translocation of QSMs across lipid bilayers. We performed umbrella sampling at atomistic resolution to calculate partitioning and translocation free energies for a set of naturally occurring QSMs, then used COSMOmic to screen the water-membrane partition and translocation free energies for 50 native and non-native QSMs that target LasR, one of the LuxR family of quorum-sensing receptors. This screening procedure revealed the influence of systematic changes to head and tail group structures on membrane partitioning and translocation free energies at a significantly reduced computational cost compared to atomistic MD simulations. Comparisons with previously determined QSM activities suggest that QSMs that are least likely to partition into the bilayer are also less active. This work thus demonstrates the ability of the computational protocol to interrogate QSM-bilayer interactions which may help guide the design of new QSMs with engineered membrane interactions.

## Introduction

Quorum sensing (QS) is a communication process that bacteria use to coordinate group behavior [1–3]. QS is regulated by the secretion and sensing of small-molecule chemical signals called quorum-sensing modulators (QSMs). When the concentration of native QSMs reaches a threshold value, QSMs can bind to and activate intracellular receptors to initiate changes in gene expression that cause bacteria to engage in group behaviors with potentially severe biomedical consequences. For example, *Staphylococcus aureus* bacteria can form multicellular aggregates, called biofilms, that are antibiotic-resistant and produce persistent infections associated with approximately twice the deaths and costs compared to typical hospitalizations [4]. Biofilm formation can further cause the failure of implanted medical devices and leads to high mortality in cystic fibrosis patients [2, 4]. QS also regulates the secretion of virulence factors that contribute to the interkingdom virulence of *Pseudomonas aeruginosa* and mutant *Escherichia coli* [5–9]. QSMs can also interact with eukaryotic hosts directly to exert immunomodulatory effects [10, 11]. Hence, there is significant interest in understanding and inhibiting QS pathways with the goal of developing therapeutic strategies to combat biofilm formation and virulence in bacteria [12–15].

Prevalent QSMs for Gram-negative bacteria include *N*-acyl homoserine lactones (AHLs) that consist of a polar homoserine lactone head group and a nonpolar aliphatic tail [13, 16]. For example, QS in *P*. *aeruginosa* is regulated by *N*-3-oxo-dodecanoyl L-homoserine lactone (3-oxo-C12-AHL) and *N*-butyryl L-homoserine lactone (C4-AHL) which bind to intracellular LuxR-type receptors. To modulate QS in these organisms, many synthetic compounds have been developed to selectively activate or inhibit QS by binding to QS receptors [17–25]. Because LuxR-type receptors are cytosolic, these compounds must bypass the cell membrane to reach the cytosol, but little is known about transport mechanisms for even naturally occurring QSMs. Most AHLs are thought to passively translocate across bacterial cell membranes [26], but some (*e*.*g*., 3-oxo-C12-AHL) are actively transported across the cell membrane via pumps [19, 26]. Some QSMs (including AHLs) have also been found to be associated with membrane vesicles that facilitate their trafficking between cells [27]. QSMs may further initiate the formation of membrane vesicles by strongly interacting with the host membrane [28]. These latter observations suggest that QSMs can partition within, rather than translocate across, the membrane. Understanding how these possible membrane interactions influence the cytosolic availability of QSMs is valuable for both understanding the activity of naturally occurring QSMs and for guiding the design of synthetic compounds capable of similar membrane interactions.

These biological observations have motivated several studies to elucidate the interactions of AHLs with model cell membranes that contain a limited number of lipid and sterol components [29]. Model cell membranes can capture the barrier properties of biological membranes without the inclusion of proteins that facilitate membrane transport, thus permitting study of partitioning and translocation processes that involve direct AHL-bilayer interactions. For example, experiments have studied the partitioning and permeation of AHLs into single- and multicomponent lipid bilayers depending on their hydrophobicity [30–33]. AHLs have also been shown to exhibit self-assembly behavior characteristic of conventional surfactants and amphiphiles [34, 35], suggesting favorable membrane partitioning. However, these techniques have only been applied to a small set of naturally occurring AHLs and are too time-consuming to broadly screen the membrane interactions of a wider range of native and non-native QSMs.

In addition to experimental studies, molecular simulations can be utilized to study the thermodynamics of membrane partitioning and translocation while providing insight into the mechanisms underlying these interactions. For example, atomistic molecular dynamics (MD) simulations, along with appropriate sampling techniques, have been used to study free energy landscapes for the partitioning and translocation of a variety of drug-like compounds, amino-acid residues, peptides, and related biomolecules [36–41]. However, atomistic MD remains too computationally demanding to apply to a broad range of QSMs. Coarse-grained MD simulations, in which multiple atoms are represented by single particles, reduce this computational expense, but require extensive parameterization and do not capture the atomistic resolution needed to resolve some molecular structures [42–44]. Alternatively, the COnductor-like Screening Model for Realistic Solvation (COSMO-RS) can be used to rapidly investigate the thermodynamics of multicomponent liquid-phase systems [45, 46]. COSMO-RS is a predictive model that combines unimolecular density functional theory calculations with statistical thermodynamics methods to account for molecular interactions and compute thermodynamic properties. The extension of COSMO-RS to anisotropic systems and membranes is called COSMOmic [47]. Given input bilayer and molecule structures generated from a short MD simulation, COSMOmic can predict the free energy for molecular partitioning into and translocating across a lipid bilayer. Prior studies have shown that such free energies computed using COSMOmic are linearly correlated with free energies calculated using atomistic MD simulations, with a particularly strong correlation obtained for partitioning free energies, and can be obtained at only a fraction of MD’s computational cost [48–51]. COSMOmic is thus a powerful tool for the high-throughput screening of QSM-membrane interactions to complement low-throughput experimental studies.

In this work, we used COSMOmic calculations in conjunction with MD simulations to analyze the partitioning and translocation of naturally occurring AHLs and synthetic QSMs (including both activators and inhibitors) across lipid bilayers. We performed umbrella sampling using atomistic MD simulations to determine the partition and translocation free energies for naturally occurring AHLs. These calculations revealed that AHLs exhibit membrane interactions similar to typical nonionic amphiphiles. We then utilized COSMOmic to predict the water-membrane partition and translocation free energies for 50 native and non-native QSMs that target LasR, one of the LuxR family of quorum-sensing receptors that binds long-tail AHLs in *P*. *aeruginosa*. COSMOmic predictions were used to reveal the influence of systematic changes to head and tail group structures on membrane partitioning and translocation. Finally, we compared simulation-derived partition free energies to experimentally measured QSM activity data and found that QSMs that are less likely to partition into the bilayer are less potent LasR activators and inhibitors. This comparison suggests that passive diffusion across the bilayer could decrease QSM activity. Together, this work demonstrates the power of the computational protocol to interrogate membrane transport pathways, which may help guide the design of new QSMs with desired membrane interactions.

## Methods

### Molecular dynamics simulations

Atomistic MD simulations were performed to obtain input structures for the COSMOmic calculations and to compute bilayer translocation free energies. QSMs and lipids were modeled using the CHARMM36 force field with the TIP3P water model [52]. Molecular structures and force field parameters were generated using the CHARMM-GUI Input Generator [53, 54]. In all MD simulations, Verlet lists were generated using a 1.2 nm neighbor list cutoff. Van der Waals interactions were modeled with a Lennard-Jones potential using a 1.2 nm cutoff that was smoothly shifted to zero between 1.0 nm and 1.2 nm. Electrostatic interactions were calculated using the smooth Particle Mesh Ewald method with a short-range cutoff of 1.2 nm, grid spacing of 0.12 nm, and 4th order interpolation. Bonds were constrained using the LINCS algorithm. All systems were energy minimized using the steepest descent method then equilibrated using a leapfrog integrator with a 2-fs timestep. The temperature was maintained at 310.15 K using a Berendsen thermostat to replicate physiological temperatures with a time constant of 1.0 ps. All MD simulations were performed using Gromacs 2016 [55].

**Fig 1:**
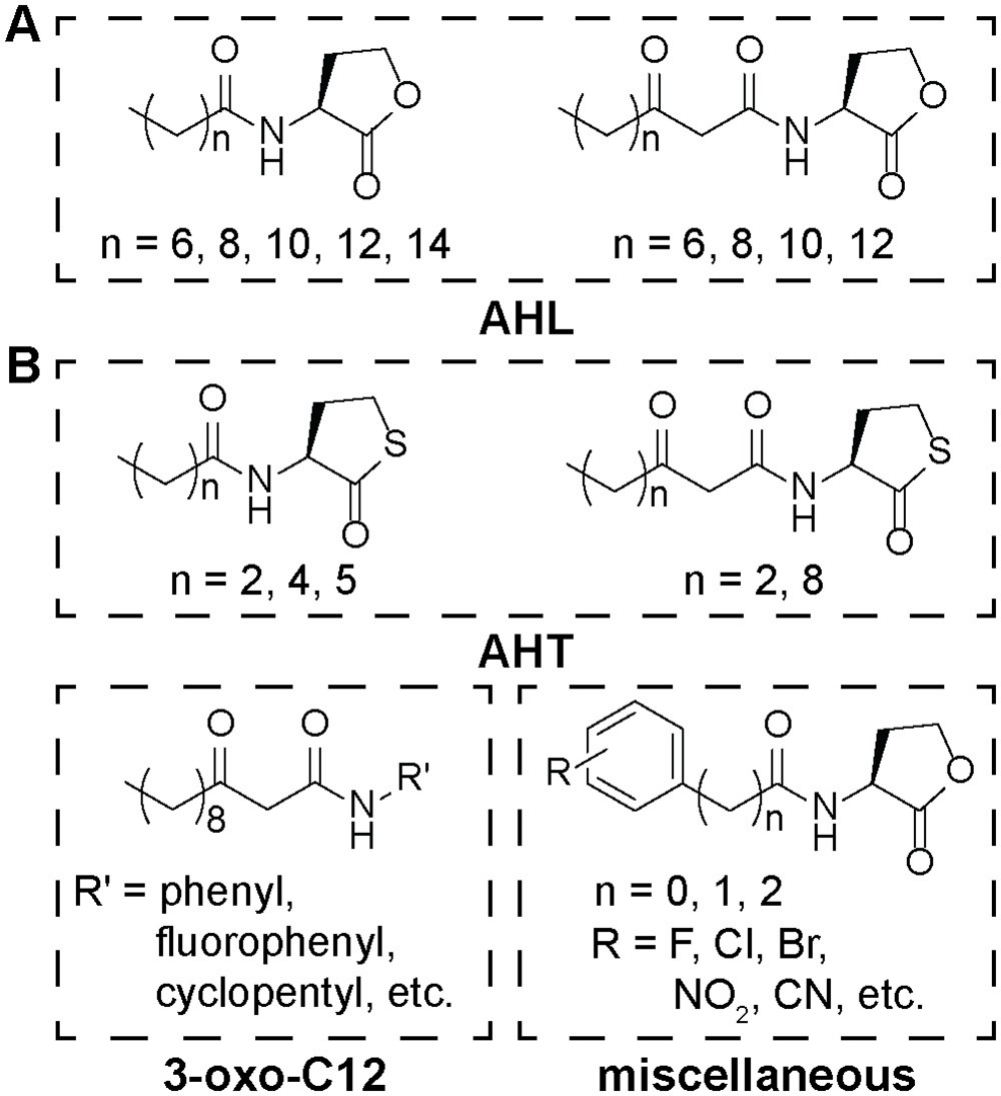
**(A)** Structures of naturally occurring *N*-acyl homoserine lactones (AHLs). **(B)** Structures of representative non-native activators and inhibitors of the quorum sensing receptor LasR (QSMs) examined in this study. The QSMs are divided into three categories: *N*-acyl homoserine thiolactones (AHTs), 3-oxo-C12 AHL derivatives (3-oxo-C12), and miscellaneous QSMs. A full list of QSMs is included in S1 Fig in S1 File.

Initial configurations of QSMs (example chemical structures in Fig 1) were solvated in water using PACKMOL [56] and equilibrated for 40 ns. The pressure was maintained at 1 bar using an isotropic Berendsen barostat with a time constant of 5.0 ps and a compressibility of 4.5×10^−5^ bar^-1^. Initial simulation configurations of a pure DOPC and 3:1 POPE/POPG lipid bilayers were generated using the CHARMM-GUI Membrane Bilayer [53, 54] with 64 lipids in each leaflet and neutralizing ions (for the anionic POPE/POPG bilayer) then equilibrated for 150 ns. This simulation time was long enough to confirm that the systems reach equilibrium [34]. The pressure was maintained at 1 bar using a semi-isotropic Berendsen barostat with a time constant of 5.0 ps and a compressibility of 4.5×10^−5^ bar^-1^.

### Free energy calculations

Molecular dynamics simulations with umbrella sampling [57] were performed to obtain potentials of mean force (PMFs) for the translocation of C4-AHL, C8-AHL, C12-AHL, and 3-oxo-C12-AHL across a DOPC bilayer. The Weighted Histogram Analysis Method (WHAM) [58] was used to calculate the PMF as a function of the *z*-component of the distance between the QSM center of mass and bilayer center of mass (denoted as *z*). Each umbrella sampling calculation was performed using independent windows separated by 0.1 nm along *z* from *z* = – 4 to 1.2 nm with *z* = 0 nm corresponding to the bilayer center. 72, 59, 57, and 68 windows were used for C4-AHL, C8-AHL, C12-AHL, and 3-oxo-C12-AHL, respectively. The number of windows was selected to obtain convergence and ensure that the PMF was symmetric around *z* = 0. Harmonic restraints with force constants ranging from 200 kJ mol^-1^ nm^-2^ to 1500 kJ mol^-1^ nm^-2^ were applied to restrain sampling to a given value of *z*. Each window was equilibrated for 5 ns in the *NPT* ensemble and then sampled for 32 ns. The last 24 ns of this trajectory were split into two independent 12-ns blocks and used to compute two PMFs using WHAM. Additional details on the umbrella sampling protocol and PMF convergence are detailed in the S1 File.

### COSMOmic calculations

**Fig 2:**
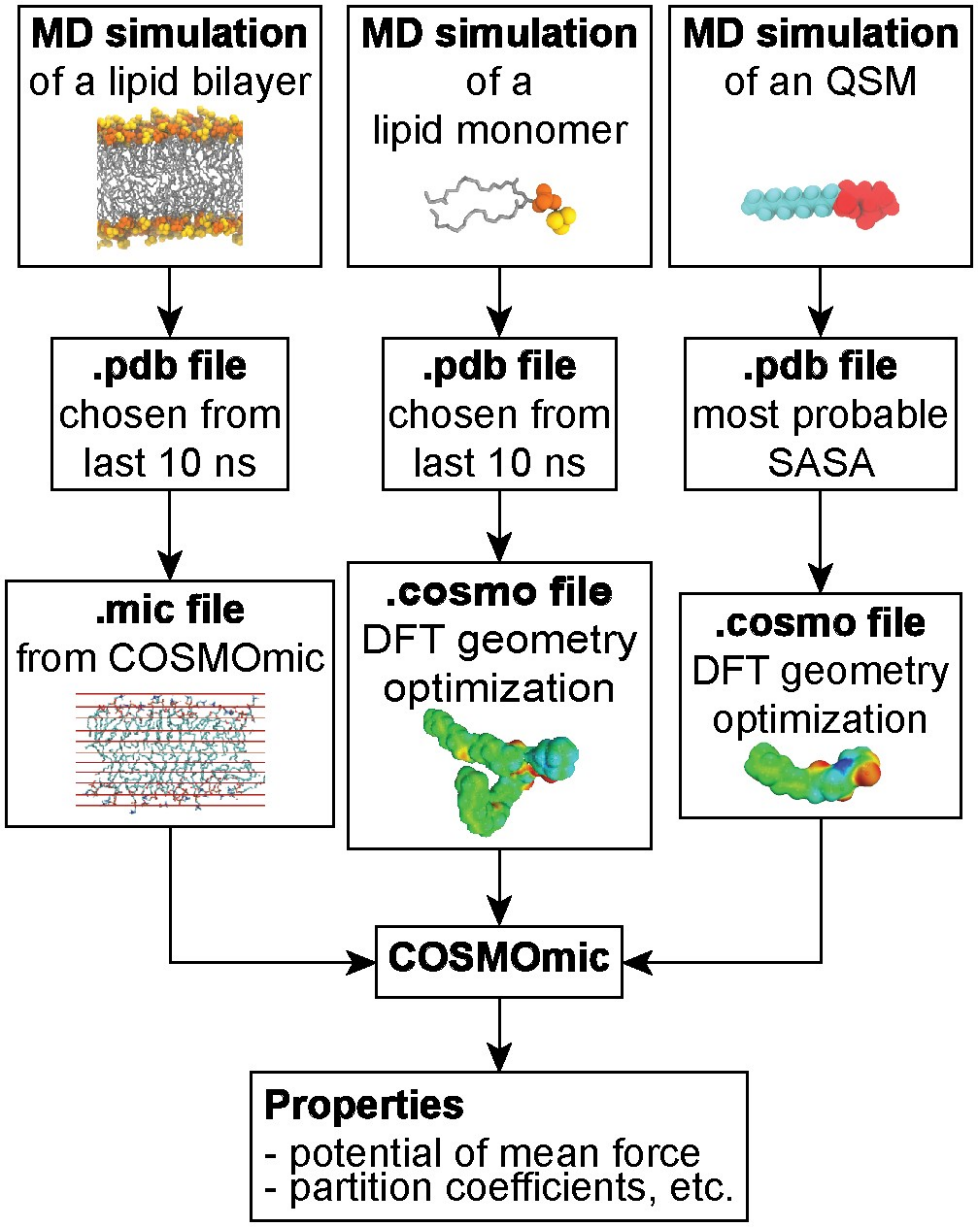
Flow diagram illustrating the inputs into COSMOmic from molecular dynamics (MD) and density functional theory (DFT) calculations that are used to predict quorum sensing modulator (QSM) interactions with lipid bilayers.

COSMOmic was used to compute PMFs for all QSMs studied in this work using the workflow summarized in Fig 2. As input, COSMOmic requires structures of the bilayer of interest (obtained from a MD simulation) and screening charge densities for each different type of molecule in the system (obtained from quantum chemistry calculations). Screening charge densities also depend on molecular configuration and thus initial configurations of molecules were generated using MD simulations. We describe each of these steps below.

Three lipid bilayer configurations were selected from the 140 ns, 144 ns and 149 ns timepoints of the atomistic MD equilibration simulation. Similarly, three lipid configurations were chosen from the 30 ns, 34 ns and 39 ns timepoints of the 40-ns simulations of individual lipids in solution. Multiple configurations were selected because prior work found that multiple configurations improve COSMOmic predictions [45]. For each QSM, configurations were selected based on the solvent-accessible surface area (SASA). The SASA of each molecule was calculated during the last 10 ns of the atomistic MD equilibration simulations using *gmx sasa* and histogrammed to compute the SASA distribution. The configuration corresponding to the most probable value of the SASA was then selected as COSMOmic input. *Gaussian16* was used to compute the screening charge densities (COSMO files) for all molecules. Geometry optimization was performed in implicit water (Conductor-like Polarizable Continuum Model, CPCM) using density functional theory at the BVP86/TZVP/DGA1 level of theory. A single point calculation was then performed to generate the ideal screening charges (at the infinite dielectric constant limit) on the molecular surface using the same level of theory [59]. Additional details on this procedure and the impact of alternative configurations are described in the S1 File.

Given the input membrane structure and screening charge densities, COSMOmic (implemented in *COSMOtherm*, version 19.0.05) divides the membrane into a series of horizontal slices and computes the water-membrane partition coefficient (*K*_*w*→*m*_) of a molecule in each slice using COSMO-RS calculations [47]. The partition coefficient is calculated by rotating each input configuration per molecule multiple times per slice, with the corresponding partition coefficient weighted by the interaction energy of each orientation. The number of orientations used did not influence the results (S2 Fig in S1 File). The partition coefficient can be related to the PMF as a function of *z* by Eq 1: [60]

$$\text{PMF}(z)=-\;RT\;\text{ln}\;K_{w\to m}(z)$$ (1)

PMF(*z*) is the free energy change for moving a molecule from a position in bulk water to the specific value of *z*, *R* is the gas constant, and *T* is the temperature. Computing *K*_*w*→*m*_ for enough values of *z* (slices) to span the bilayer thus yields the PMF that can be compared to umbrella sampling calculations. 20 slices were used to span the membrane, leading to a spacing of approximately 0.2 nm [45]. S3 Fig in S1 File shows that the number of slices does not substantially influence the resulting PMFs.

## Results and discussion

### Partition and translocation free energies for natural AHLs

**Fig 3:**
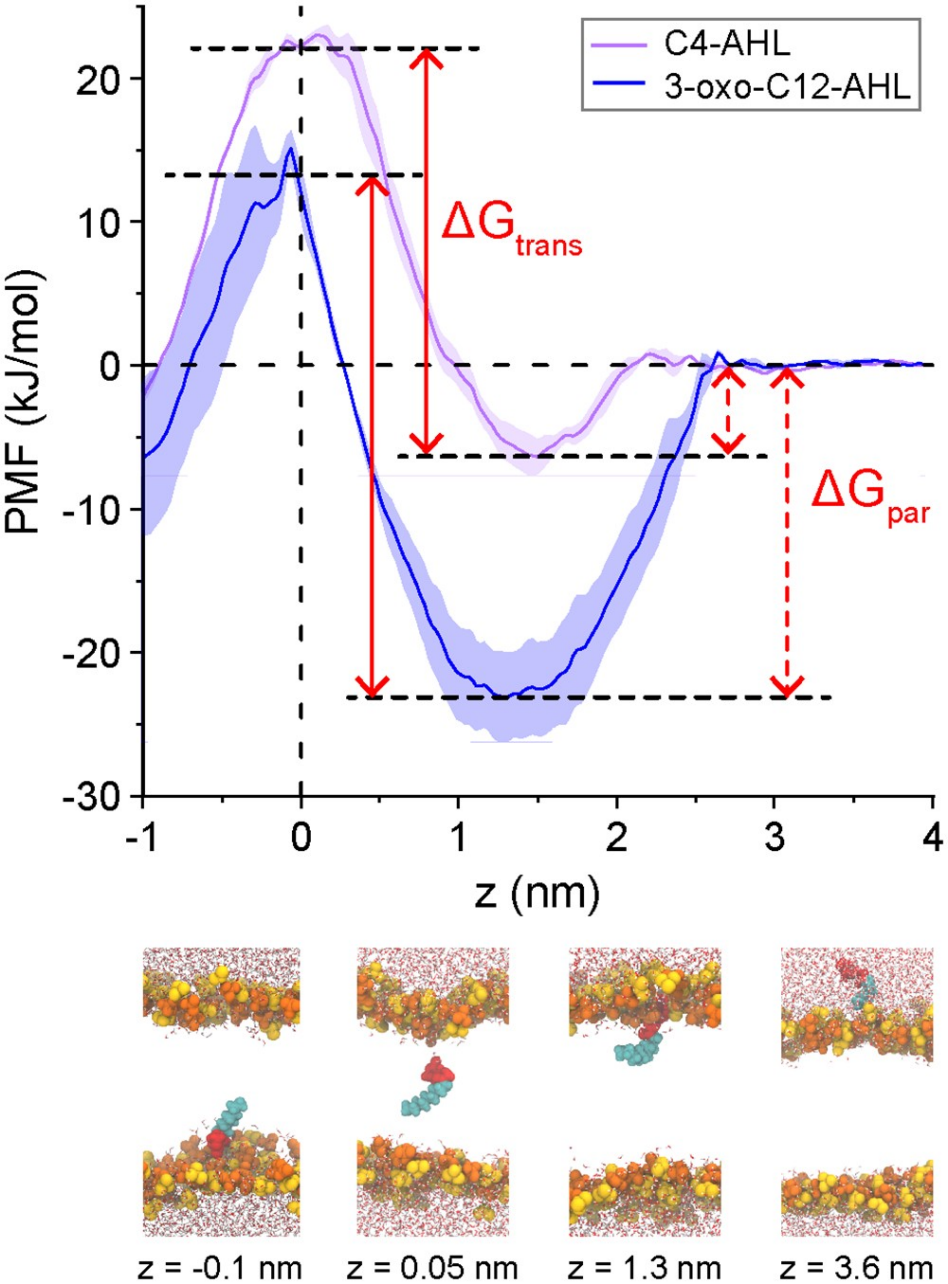
Potentials of mean force (PMFs) of C4-AHL and 3-oxo-C12-AHL as a function of the distance along the DOPC bilayer normal (*z*). PMFs were computed using umbrella sampling with the PMF set to zero at *z* = 4 nm. Each curve shows the average of two independent PMFs and error bars shows the standard deviation. The midplane of the membrane is at z = 0 nm. The plots also illustrate the definition of ΔG_par_ as the free energy difference between the PMF minimum and the value in solution and ΔG_trans_ as the free energy difference between the PMF minimum and maximum. Snapshots at bottom illustrate the configurations of AHLs and bilayers for different characteristic values of *z* in the PMF of 3-oxo-C12-AHL. Lipid head groups are yellow/orange, AHL head groups are red, AHL tail groups are cyan, and water is drawn as red/white. Lipid tail groups are not shown for clarity.

We first calculated potentials of mean force (PMFs) for the translocation of C4-AHL and 3-oxo-C12-AHL (Fig 1) across a single-component DOPC lipid bilayer using umbrella sampling. DOPC was selected as a typical zwitterionic lipid which is in the fluid phase in physiological conditions and has been used experimentally in model membranes [29]. The PMFs quantify the change in free energy associated with translating each AHL along the reaction coordinate relative to a reference value set to zero at *z* = 4 nm, which corresponds to a position in solution away from the bilayer. Fig 3 shows the PMFs along with representative simulation snapshots illustrating molecular configurations for different values of *z*. Both PMFs are approximately symmetric around the bilayer midplane (at *z* = 0 nm), as expected given the symmetry of the system, indicating that the PMFs are converged. There is a slight asymmetry in the 3-oxo-C12-AHL PMF which likely results from the deformation of the bilayer as illustrated in the inset snapshot. Such deformations can introduce hysteresis leading to PMF asymmetry [61]. Nonetheless, the plateau value of the PMF near *z* = 0 nm indicates that the value at the PMF maximum is converged.

PMFs for both AHLs exhibit small (~1 kJ/mol) local maxima, located at *z* = 2 nm for C4-AHL and *z* = 3 nm for 3-oxo-C12-AHL. PMF maxima correspond to free energy barriers, which in this case arise due to the slight deformation of lipid head groups as the AHLs begin to partition into the membrane. As *z* decreases, both PMFs exhibit global minima near *z* = 1.2 nm that correspond to the insertion of the nonpolar tail group of the AHL into the bilayer core with the head group remaining near the bilayer-water interface. Finally, both PMFs exhibit global maxima at *z* = 0 nm, which is the bilayer midplane. In this configuration, the bilayer is substantially deformed to minimize the exposure of the AHL head group to the nonpolar lipid tails. These features are generally consistent with prior PMFs computed for amphiphiles [48, 62, 63] and confirm that AHLs exhibit amphiphilic behavior as indicated by prior simulations and experiments [30, 34, 35]. Similar features were also observed for two additional naturally occurring AHLs, C8-AHL and C12-AHL (S4 Fig and S2 Table in S1 File), that vary in tail group length.

Based on the PMFs, we define two free energy differences relevant to QSM-bilayer interactions (schematically illustrated in Fig 3). The difference in free energy between the PMF minimum and the reference state in solution is defined as the partition free energy (ΔG_par_). ΔG_par_ captures the favorable partitioning of QSMs into the bilayer, driven by hydrophobic interactions between nonpolar groups and lipid tail groups. Fig 3 shows that ΔG_par_ is more negative for 3-oxo-C12-AHL (-23.16 ± 3.13 kJ/mol) than C4-AHL (-6.36 ± 1.22 kJ/mol) due to the longer tail group of 3-oxo-C12-AHL. The larger magnitude of ΔG_par_ for 3-oxo-C12-AHL is consistent with experimental observations that this AHL accumulates within the membrane, as opposed to C4-AHL which passively diffuses across the membrane [26]. These values are also consistent with partition free energies for drug-like nonionic amphiphiles in DOPC membrane, for which ΔG_par_ ranges between -7.5 to -100 kJ/mol [26]. The difference in free energy between the PMF maximum and the PMF minimum is defined as the translocation free energy (ΔG_trans_). ΔG_trans_ is the free energy barrier for crossing the membrane that emerges from the significant deformation of the bilayer to avoid contact between polar groups and nonpolar lipid tails. ΔG_trans_ was 28.63 ± 1.23 kJ/mol for C4-AHL and 35.18 ± 3.37 kJ/mol for 3-oxo-C12-AHL, indicating that both AHLs experience barriers for crossing the bilayer. These values are again comparable to those of drug-like nonionic amphiphiles, for which ΔG_trans_ ranges from 0 to 60 kJ/mol [26]. The slightly smaller barrier for C4-AHL suggests that diffusion across the bilayer occurs more readily than for 3-oxo-C12-AHL, which is consistent with experimental observations of passive diffusion of C4-AHL [26]. The higher barrier for 3-oxo-C12-AHL may also explain why the molecule is not observed to flip-flop in supported lipid bilayers [32].

### Comparison of potentials of mean force from MD and COSMOmic

The PMFs generated from MD with umbrella sampling are in good agreement with prior simulation studies and known experimental observations of AHL behavior, but these calculations are time-consuming, requiring approximately 1.5 μs of atomistic MD simulation time per PMF. This computational expense inhibits the screening of bilayer interactions across a wider range of QSMs. Alternatively, COSMOmic is a computationally efficient tool to enable the high-throughput calculation of bilayer partition and translocation free energies, requiring approximately 500 times less computational time than the umbrella sampling calculations. Therefore, we sought to compare COMSOmic predictions to the umbrella sampling results to determine if COSMOmic could be used to systematically investigate the effect of QSM properties on their bilayer interactions.

**Fig 4:**
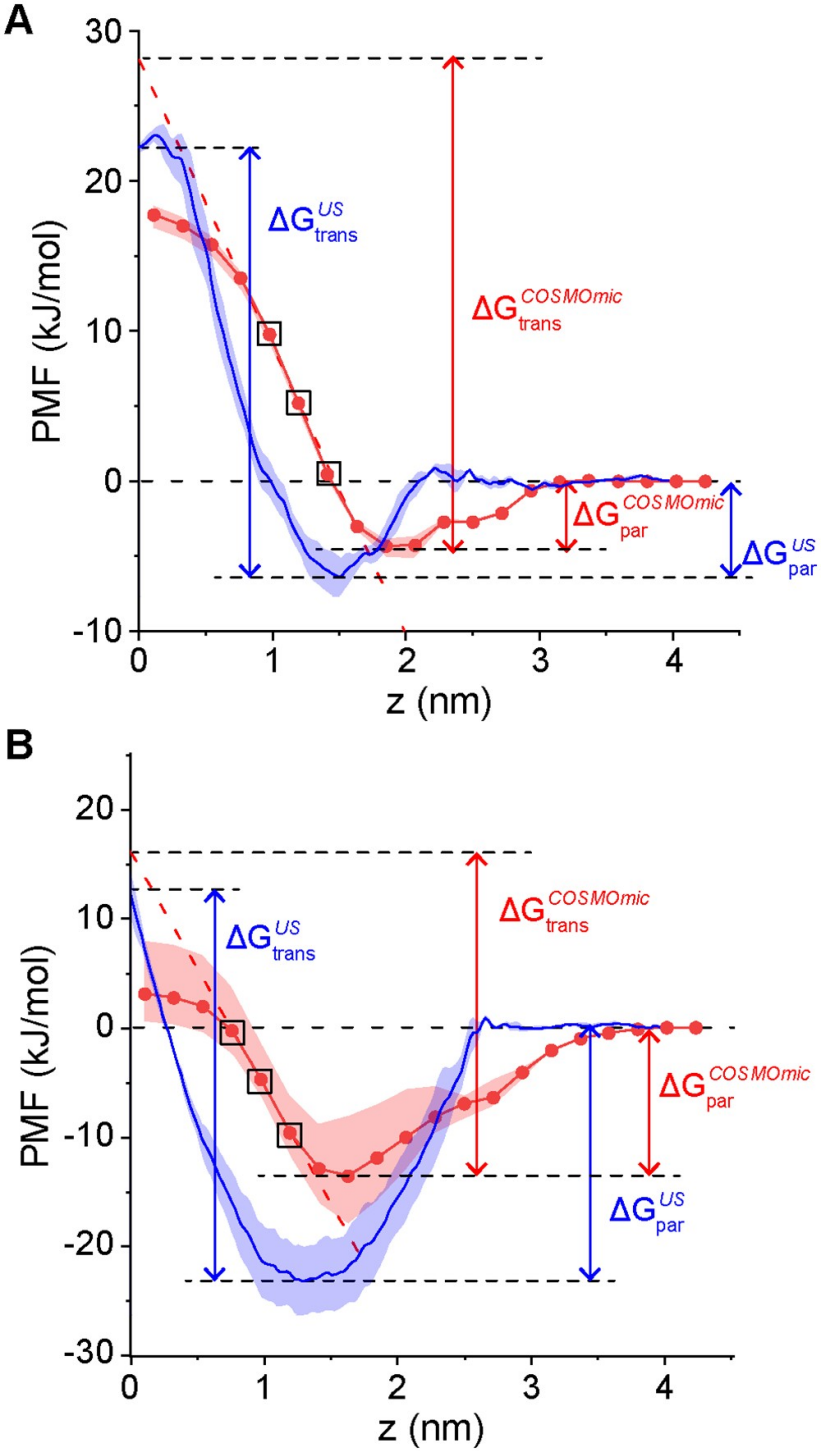
Potentials of mean force (PMFs) of A) C4-AHL and B) 3-oxo-C12 AHL as a function of the distance along the DOPC bilayer normal (*z*). PMFs computed from molecular dynamics simulations with umbrella sampling (blue; reproduced from Fig 3) are compared to PMFs computed with COSMOmic (red). ΔG_par_ and ΔG_trans_ are defined as in Fig 3 and labelled. ΔG_trans_ for the COSMOmic PMFs is determined by linear extrapolation of the points in black boxes to *z* = 0. Error bars indicate the standard deviation between three simulation replicas.

Fig 4 compares PMFs computed using COSMOmic to PMFs computed using umbrella sampling (reproduced from Fig 3). COSMOmic correctly reproduces the two primary features of the PMFs: a free energy minimum near the bilayer-water interface that defines ΔG_par_ and a free energy maximum at the bilayer midplane that defines ΔG_trans_. Compared to the PMFs from umbrella sampling, several differences are noted. First, the COSMOmic PMFs do not capture the small (~1 kJ/mol) local maxima observed near *z* = 2 nm. Because this maximum is generally smaller than thermal energy, it is not expected to influence system behavior. Second, the minima of the COSMOmic PMFs are shifted to larger values of *z* and larger PMF values that the umbrella sampling PMFs, indicating that COSMOmic underpredicts ΔG_par_ compared to the MD calculations. Past results have similarly shown that COSMOmic systematically underpredicts MD-derived partition free energies but the predicted values from both approaches are linearly correlated [48, 51], indicating that COSMOmic correctly captures trends that enables the comparison between partitioning of various species. Finally, the COSMOmic PMFs plateau near the bilayer midplane whereas the umbrella sampling PMFs increase to a maximum. This behavior was again previously reported in prior literature [48–50] and we attribute it to the inability of COSMOmic to capture bilayer deformations observed in the MD simulations.

Prior studies used the free energy difference between the PMF minimum and *z* = 0 nm to compute ΔG_trans,_ but this method systematically underestimates values from umbrella sampling due to the plateau [51]. Hence, we linearly extrapolated the PMF using the third, fourth and fifth closest points to *z* = 0 (where the PMF decreased most steeply, points in black boxes), and ΔG_trans_ was defined as the free energy difference between the minimum and the intercept of the extrapolated line, leading to better agreement with the umbrella sampling calculations in this work (S2 Table in S1 File). S5 Fig in S1 File shows that there is a strong linear correlation between ΔG_trans_ computed using linear extrapolation and the difference between the PMF minimum and z = 0 nm, indicating that either method would produce similar qualitative trends. S2 Table in S1 File compares values of ΔG_par_ and ΔG_trans_ computed using umbrella sampling and COSMOmic for the C4-AHL, C8-AHL, C12-AHL, and 3-oxo-C12-AHL. These comparisons, along with similar comparison for drug-like molecules in past work [48, 51], indicate that COSMOmic calculations do not quantitatively reproduce the values of ΔG_par_ and ΔG_trans_ calculated using atomistic MD but do capture trends in the relative values of these free energies. In particular, COSMOmic accurately predicts trends in ΔG_par_ [48, 51] which is the free energy that differs most substantially between C4-AHL and 3-oxo-C12-AHL (Fig 3) and may determine their distinct intracellular availability.

### Effect of AHL head and tail group properties on bilayer interactions

**Fig 5:**
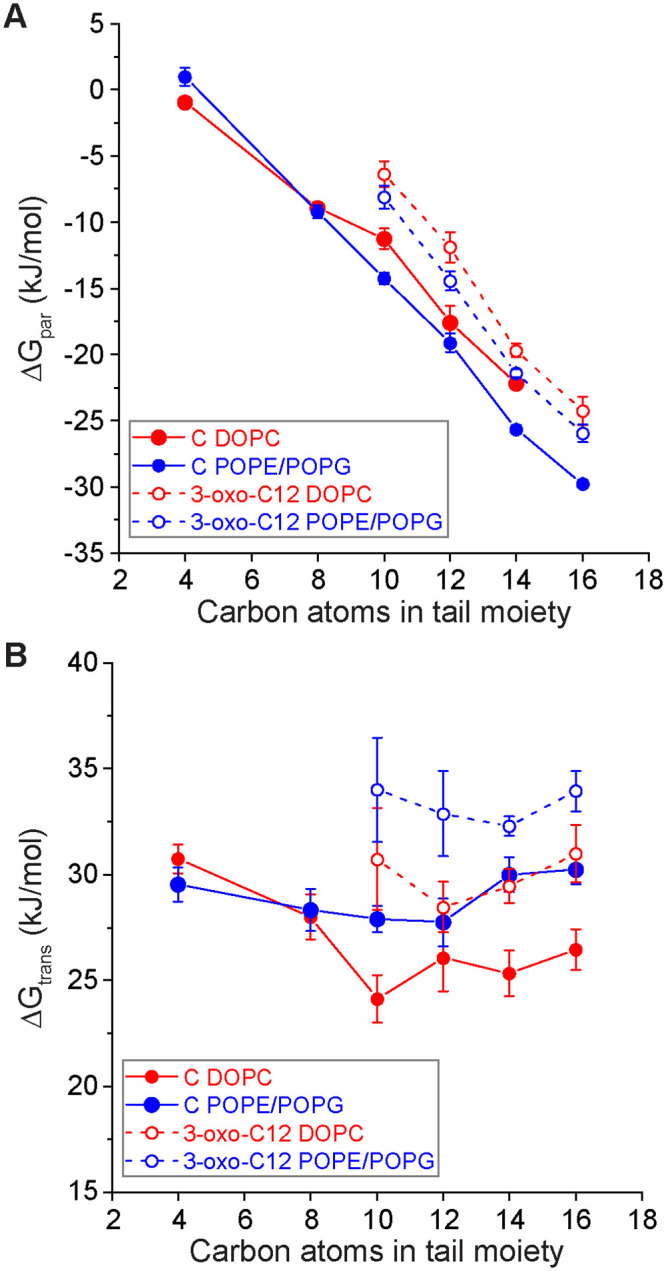
ΔG_par_ (A) and ΔG_trans_ (B) for AHL interactions with DOPC (red) and POPE/POPG (blue) bilayers. Hollow and filled circles indicate AHLs with and without the 3-oxo group in the head moiety, respectively. Error bars represent the standard error from three independent calculations using different input conformations to COSMOmic.

Using COSMOmic calculations, we next investigated trends in the partitioning and translocation of naturally occurring AHLs in single-component DOPC and multicomponent POPE/POPG bilayers. The POPE/POPG mixture was selected to mimic the lipid composition of a bacterial inner cell membrane, and notably differs from DOPC because POPG head groups are anionic. Fig 5 compares ΔG_par_ and ΔG_trans_ for 9 AHLs of varying head and tail functionalities for both bilayer compositions. There were no substantial differences in these free energies between DOPC and POPE/POPG membranes; therefore, we only used neutral DOPC bilayers for the remainder of this study. This finding also supports the use of a single-component DOPC bilayer in experiments as a model membrane for multiple cell types to capture bilayer interactions [32].

Fig 5 shows that ΔG_par_ is dictated by the AHL tail length, with longer tails leading to more negative values of ΔG_par_ indicating more thermodynamically favorable partitioning. This result is consistent with typical studies of amphiphile partitioning into lipid bilayers, further emphasizing that long-tail AHLs act like amphiphiles [48, 62, 63]. 3-oxo-C12-AHL and C16-AHL, which have been experimentally found to associate with bilayers [27, 32], both have large, negative values of ΔG_par_ in agreement with these expectations. AHLs with a 3-oxo head group have a systematic increase of ~5 kJ/mol to ΔG_par._ This result is similar to the prior observation of a systematic increase in the critical micelle concentrations of AHLs with 3-oxo groups compared to AHLs lacking 3-oxo groups [34]. Values of ΔG_trans_ were only modestly influenced by the AHL tail length but were influenced by the presence of a 3-oxo moiety, which tended to increase ΔG_trans_. These general trends are consistent with the 3-oxo group increasing the hydrophilicity of the AHL head group and more carbon atoms providing a stronger hydrophobic driving force for insertion, with the head group dictating the translocation barrier.

### Partitioning and translocation free energies for non-native QSIs

**Fig 6:**
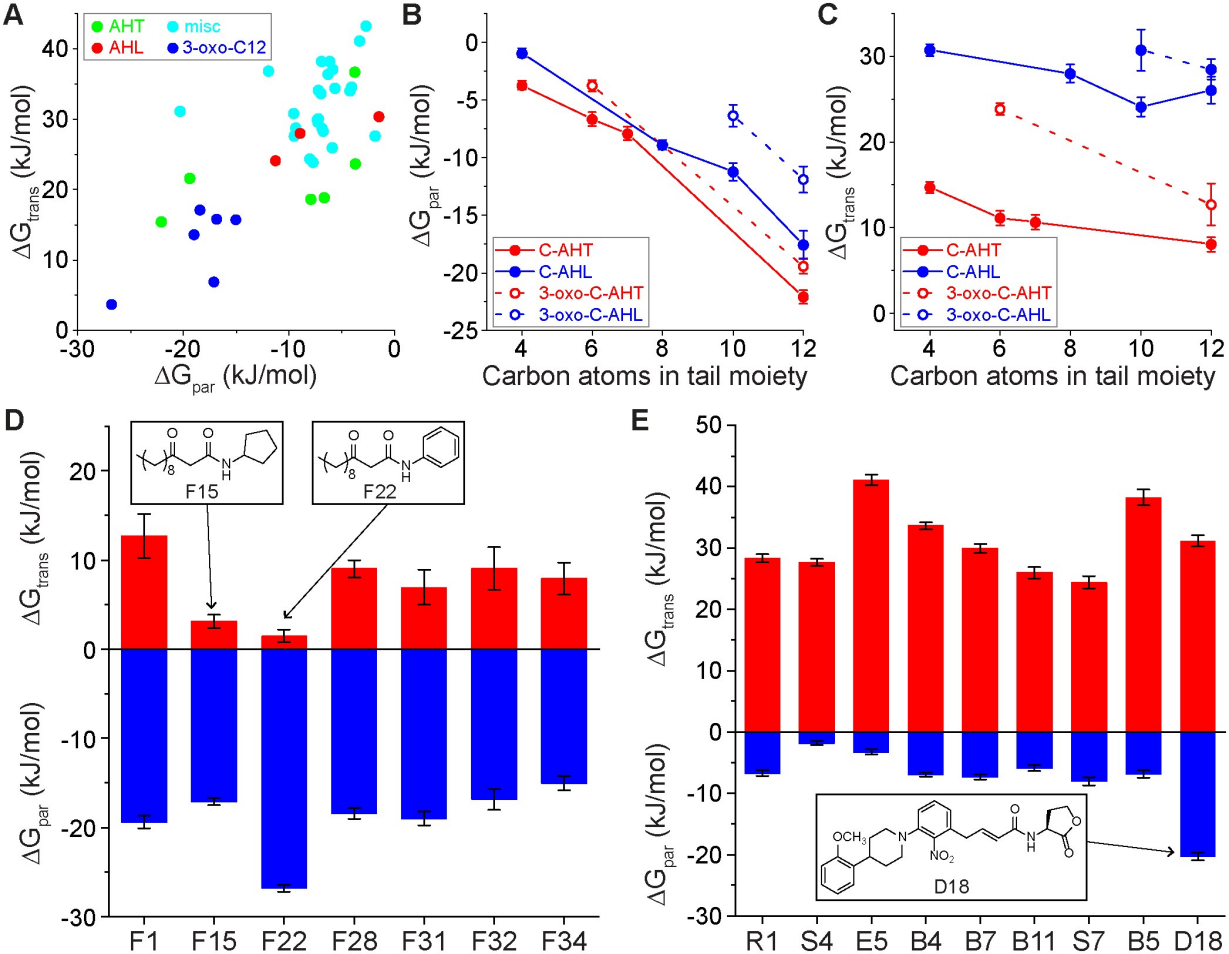
**(A)** ΔG_par_ and ΔG_trans_ for four categories of QSMs. **(B)** ΔG_par_ for *N*-acyl homoserine thiolactones (AHTs) and AHLs. The hollow and filled markers represent AHLs with and without the oxo additional group in the head moiety, respectively. **(C)** ΔG_trans_ for AHT and AHLs. **(D)** ΔG_par_ and ΔG_trans_ for 3-oxo-C12 derivatives. **(E)** ΔG_par_ and ΔG_trans_ for miscellaneous QSMs. Error bars in all parts represent the standard error from three independent calculations using different input conformations to COSMOmic.

The results from Figs 4 and 5 provide bounds on the partition and translocation free energies expected for naturally occurring AHLs, including AHLs with known experimental behavior. We next extended the COSMOmic calculations to a group of 40 non-native QSMs that target intracellular LasR receptors. Prior experimental studies have quantified the ability of these molecules to activate or inhibit quorum sensing [18–20, 22–25, 64, 65]; we sought to quantify their membrane interactions (Fig 6). QSMs were divided into four general categories: AHLs (C8-AHL, C10-AHL, and 3-oxo-C6-AHL, because these are LasR inhibitors), AHTs, 3-oxo-C12 derivatives, and miscellaneous (“misc”). These molecules possess a diverse range of structures and chemical groups to permit analysis of how such features influence membrane interactions. Representative molecular structures are shown in Fig 1 with all chemical structures shown in S1 Fig in S1 File. Naming conventions follow the conventions used in prior literature for each set of molecules [18–20, 22–25, 64, 65]. Fig 6A plots ΔG_par_ and ΔG_trans_ for all QSMs studied. Points are color-coded according to the four categories described above. A moderate positive linear correlation was found between ΔG_par_ and ΔG_trans_ across all QSMs, indicating that for these molecules stronger partitioning into the bilayer (more negative value of ΔG_par_) inhibits translocation (more positive value of ΔG_trans)_. Values for both free energies span a wide range of values within a similar range as the reference naturally occurring AHLs. We thus further investigated how variations in chemical structure influenced membrane interactions for each QSM category.

Fig 6B compares ΔG_par_ for the set of AHTs as a function of the AHT tail length. AHTs are similar to AHLs but bear a thiolactone head group. Like the AHLs, increasing AHT tail length led to more favorable partitioning, with an additional 3-oxo group in the head group decreasing the magnitude of ΔG_par_. The AHT head group led to slightly stronger partitioning compared to the AHL head group. Fig 6C further compares ΔG_trans_ as a function of AHT tail length. In general, ΔG_trans_ was smaller for AHTs than AHLs of the same tail length and was more strongly dependent on the tail length. These data indicate that the thiolactone head group causes AHTs to appear more hydrophobic than AHLs due to a single atom substitution.

We next investigated the set of 3-oxo-C12 derivatives, which had identical tail functionalities as 3-oxo-C12-AHL but varied in the head group chemistry. Fig 6D shows that values of ΔG_par_ are similar for these molecules, likely due to the identical tail structure in each. However, molecule F22 (with a phenyl head group) had a more favorable partition free energy than the rest of the set. We attribute this difference to the preference of aromatic head groups for the bilayer-water interface, which could stabilize partitioning. Overall, however, the similar values of ΔG_par_ across this set of molecules suggests that this parameter can be tuned by tail properties nearly independently of the head group. Unlike ΔG_par_, Fig 6D shows that values of ΔG_trans_ substantially vary across the set of molecules. Notable examples with unusually low values of ΔG_trans_ are F15 (with a cyclopentyl head group) and F22. These molecules were unique in that their hydrocarbon tails were connected to the head groups via peptide bonds, which implies that this functionality decreases the translocation free energy barrier. In the case of F15, this decrease is not accompanied by a change in ΔG_par_. Together, these results indicate the ability of minor chemical modifications to lead to separate variations to ΔG_par_ and ΔG_trans_, potentially enabling control over the preferential diffusion or accumulation of QSMs in bilayers.

Finally, Fig 6E shows ΔG_par_ and ΔG_trans_ for the set of miscellaneous QSMs. Molecule D18 was an outlier in ΔG_par_ because it had the longest tail group among the set. In general, values of ΔG_par_ for miscellaneous representative QSMs were generally closer to that of C4-AHL than 3-oxo-C12-AHL since those molecules were relatively small, suggesting that the size or length of the molecules can determine the value of ΔG_par_. Similarly, ΔG_trans_ for those molecules was similar to both C4 and 3-oxo-C12-AHL. These results indicate that variations to ring structures and the location of specific groups did not substantially influence bilayer interactions compared to the length of the tail group.

**Fig 7:**
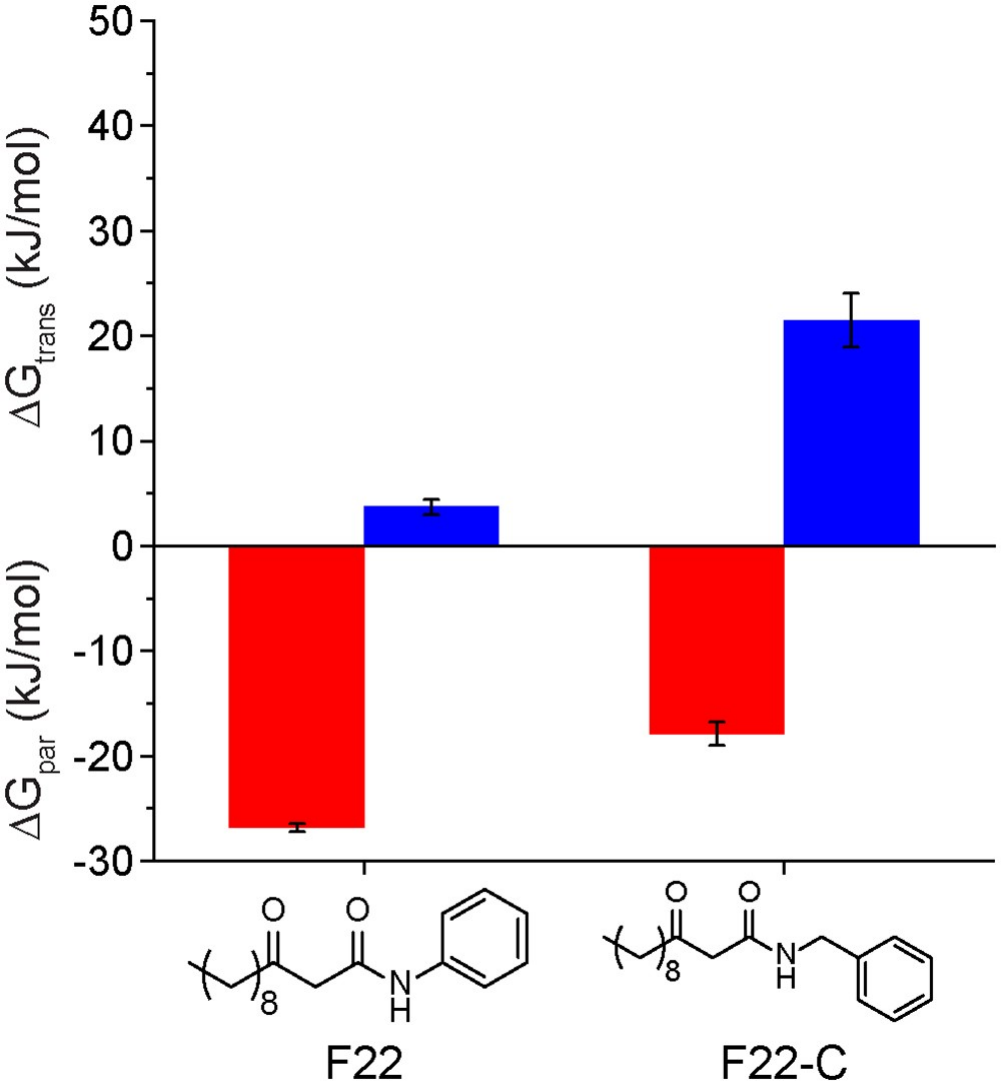
ΔG_par_ and ΔG_trans_ for F22 and F22-C (with an added methylene group).

Based on the analysis of the full set of QSMs, we sought to determine if variations in QSM chemical structures could lead to predictable effects on ΔG_par_ and ΔG_trans_. One noteworthy finding from the prior data was the behavior of the F22 molecule, which contained a phenyl head group connected via a peptide bond to a hydrocarbon tail. Compared to other 3-oxo-C12 QSIs, F22 had a significantly lower value of ΔG_par_ and lower value of ΔG_trans_. To test whether this effect was due to the inclusion of the aromatic head group, we computed ΔG_par_ and ΔG_trans_ for an artificial molecule (F22-C) with an additional methylene group inserted between the peptide bond and aromatic ring. Fig 7 shows that this modification raised both values to be comparable to the rest of the molecules. This example demonstrates how COSMOmic calculations could help guide the design of QSMs with tailored membrane interactions.

### Relationship between membrane interactions and QSM activity

Finally, we sought to determine if there is relationship between QSM-bilayer interactions (quantified via ΔG_par_) and experimental metrics quantifying LasR activation and inhibition reported in past literature [18–20, 22–25, 64–66]. Since Figs 3 and 5 indicate that the primary difference between C4-AHL and 3-oxo-C12-AHL is the value of ΔG_par_, we hypothesized that bilayer interactions could influence the localization of QSMs to either the cell interior (for molecules with ΔG_par_ similar to C4-AHL) or to membranes (for molecules with ΔG_par_ similar to 3-oxo-C12-AHL), which might then influence intracellular QSM concentrations and QSM-receptor binding. QSM activity was quantified by two metrics obtained from published, experimentally determined dose-response curves: the EC_50_, which is the QSM concentration that induces 50% of the maximum response for QSMs that activate the LasR receptor, and the IC_50_, which is the QSM concentration that inhibits 50% of the response for QSMs that inhibit the LasR receptor. EC_50_ and IC_50_ values were determined for mutant strains of *E*. *coli* that expressed the LasR receptor. IC_50_ values were determined in the presence of a concentration of 3-oxo-C12-AHL (the native activator of LasR) equal to its EC_50_. IC_50_ values were also obtained for a mutant reporter strain of *P*. *aeruginosa* that does not produce native AHLs. Tabulated EC_50_ and IC_50_ values and corresponding references are listed in S3 Table in S1 File.

**Fig 8:**
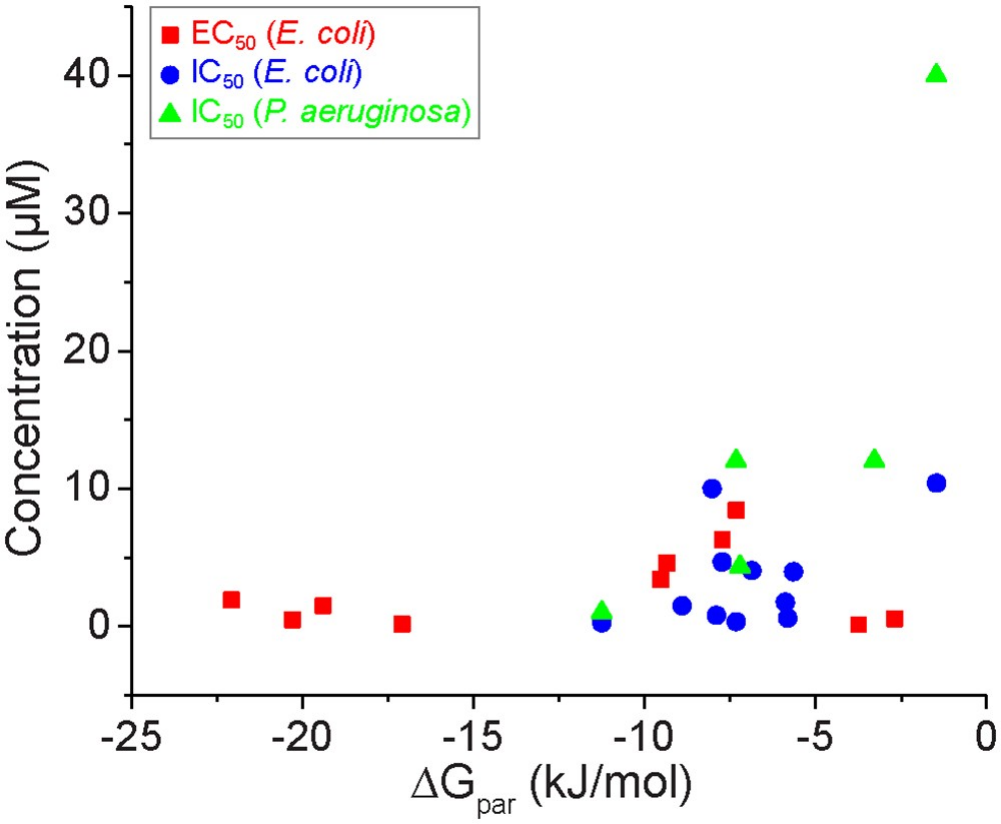
Relationship between experimental measurements of LasR activation (EC_50_) and inhibition (IC_50_) and ΔG_par_. Experimental data are taken from past literature Inhibition experiments were conducted in either *E*. *coli* or *P*. *aeruginosa* whereas activation experiments were performed in *E*. *coli*.

Fig 8 compares EC_50_ values in *E*. *coli* and IC_50_ values in both *E*. *coli* and *P*. *aeruginosa* to values of ΔG_par_ computed using COSMOmic. For comparison, ΔG_par_ is -11.90 ± 1.12 kJ/mol for 3-oxo-C12-AHL and -0.94 ± 0.40 kJ/mol for C4-AHL (Fig 5). In general, less negative values of ΔG_par_ correspond to larger EC_50_ and IC_50_ values in both *E*. *coli* and *P*. *aeruginosa*, although compounds B2 (ΔG_par_ = -2.70 kJ/mol) and F3 (ΔG_par_ = -3.75 kJ/mol) have small EC_50_ values. Notably, EC_50_ and IC_50_ values increase for QSMs with values of ΔG_par_ larger than -10 kJ/mol. The decrease in EC_50_ and IC_50_ values for QSMs with values of ΔG_par_ more similar to 3-oxo-C12-AHL than C4-AHL suggests that these compounds may readily associate with the membrane due to their favorable partitioning free energies. Intracellular concentrations of 3-oxo-C12-AHL have also been shown to exceed extracellular concentrations by a factor of 3 due to membrane association [26], which could explain the increased potency of QSMs in Fig 8 that strongly partition into the bilayer. In contrast, the passive diffusion of QSMs with values of ΔG_par_ similar to C4-AHL could lead to reduced intracellular concentrations that limit potency. Supporting this idea, prior results have shown that active efflux pumps that reduce the intracellular concentration of AHLs decrease their potency as activators [19]. Together, these behaviors may explain why bilayer interactions (quantified by ΔG_par_) relates to QSM potency across two organisms (*E*. *coli* and *P*. *aeruginosa*), both LasR activators and inhibitors, and a variety of QSM chemical structures. However, we do caution that ΔG_par_ values could also reflect, in part, the binding of the QSMs to the LasR receptor, and thus further experimental studies are needed to fully disentangle effects due to receptor and bilayer interactions.

## Conclusions

In this work, we used a combination of COSMOmic and atomistic molecular dynamics simulations to investigate lipid bilayer interactions for 10 naturally occurring AHLs and 40 quorum sensing modulators. We performed umbrella sampling calculations to compute water-membrane partition and translocation free energies for C4-AHL and 3-oxo-C12-AHL which represent naturally occurring QSMs. These AHLs exhibit bilayer interactions typical of nonionic amphiphiles, exhibiting both local free energy minima indicative of favorable bilayer partitioning and global free energy maxima indicative of barriers to translocation. 3-oxo-C12-AHL partitions strongly into the lipid bilayer whereas C4-AHL only weakly into the bilayer, agreeing qualitatively with experimental studies indicating that 3-oxo-C12-AHL associates with bilayers while C4-AHL passively diffuses across the bilayer. Using COSMOmic, we further screened partition and translocation free energies as a function of AHL tail length in both DOPC and mixed DOPG/DOPC bilayers. We found that increasing the tail length drove more favorable partitioning but the barrier to translocation was only affected by the AHL head group, with the 3-oxo group leading to a slight increase in the translocation barrier. We then systematically investigated the effect of head and tail group structures on membrane partitioning and translocation for 40 QSMs that have been experimentally shown to activate or inhibit the intracellular LasR receptor, providing insight into how variations in QSM chemical structures influence bilayer interactions. By comparing to experimental measurements of LasR activation and inhibition, we showed that QSMs which weakly partition into the bilayer (and thus are expected to behave similarly to C4-AHL) are less potent than QSMs which strongly partition into the bilayer (and thus are expected to behave similarly to 3-oxo-C12-AHL). Together, these findings provide new insight into how the structure of QSM head and tail groups affects their partitioning and translocation across the lipid bilayer which can help to provide information on membrane transport to complement experimental studies of QSM potency.

## Supporting information

S1 File (PDF)
