# Supplementary material for: Molecular simulations of lipid membrane partitioning and translocation by bacterial quorum sensing modulators
Source: PLoS One. 2021 Feb 9;16(2):e0246187. doi: 10.1371/journal.pone.0246187 (PMC7872223; doi:10.1371/journal.pone.0246187)
Supplement: S1 File — (PDF) [file pone.0246187.s001.pdf]

S1 File

Molecular Simulations of Lipid Membrane Partitioning and Translocation by Bacterial Quorum Sensing Modulators

Tianyi Jin, Samarthaben J. Patel, and Reid C. Van Lehn*

Department of Chemical and Biological Engineering
University of Wisconsin – Madison, Madison, WI, 53706, USA.

*send correspondence to: vanlehn@wisc.edu

Contents.

Figure S1. Structures of non-native LasR quorum sensing modulators.

Table S1. Comparison of COSMOmic free energies computed for different molecular conformations.

Figure S2. Potentials of mean force (PMFs) computed using COSMOmic for 3-oxo-C12-AHL as a function of the distance, *z*, along the DOPC membrane normal with different numbers of molecular orientations.

Figure S3. Potentials of mean force (PMFs) computed using COSMOmic for 3-oxo-C12-AHL as a function of the distance, *z*, along the DOPC membrane normal with different numbers of membrane slices.

Figure S4. Convergence of the potentials of mean force (PMFs) computed using umbrella sampling as a function of the distance, *z*, along the DOPC membrane normal.

Table S2. Comparison of partition (ΔG_{par}) and translocation (ΔG_{trans}) free energies calculated using umbrella sampling and COSMOmic for C4-AHL, C8-AHL, C12-AHL and 3-oxo-C12-AHL.

Figure S5. Comparison of translocation free energies (ΔG_{trans}) computed using COSMOmic determined either directly from the PMF (*x*-axis) or by linear extrapolation (*y*-axis).

Table S3. Experimental measurements of LasR activation (EC_{50}) and inhibition (IC_{50}) and corresponding values ΔG_{par} and ΔG_{trans} for various quorum sensing modulators.

References

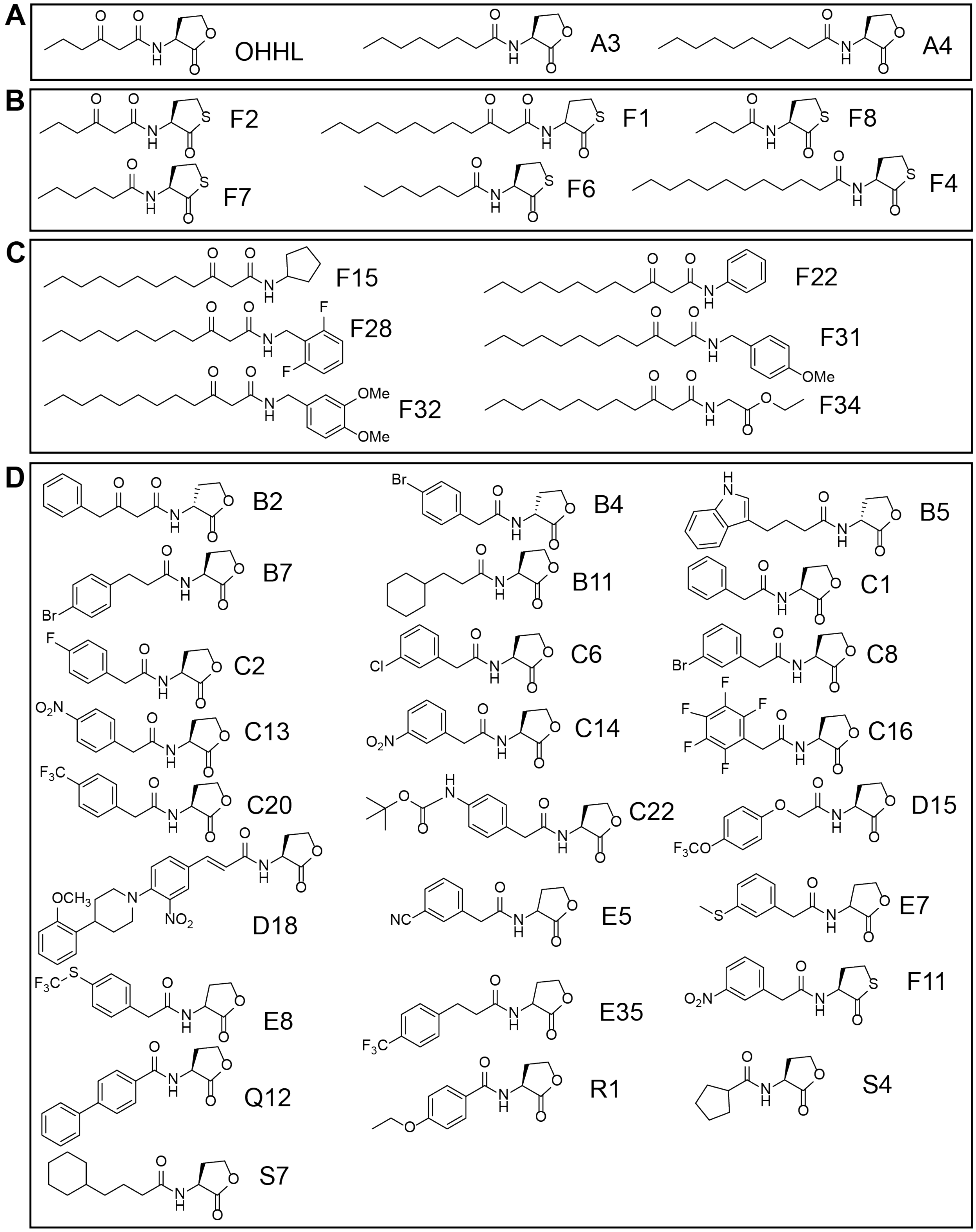

Figure S1. Structures of non-native LasR quorum sensing modulators. **(A)** N-acyl homoserine lactones (AHLs), **(B)** N-acyl homoserine thiolactones (AHTs), **(C)** 3-oxo-C12 QS inhibitors and **(D)** miscellaneous.

Comparison of COSMOmic parameters

As described in the main text, the QSM conformation used to perform COSMOmic calculations was selected based on the solvent-accessible surface area (SASA) calculated from a molecular dynamics (MD) simulation. Table S1 compares partition ΔG_{par}) and translocation (ΔG_{trans}) free energies computed using COSMOmic for different QSM conformations. Selected conformations were either the conformation corresponding to the largest SASA ("Max SASA"), the conformation with a SASA closest to the ensemble-averaged SASA ("Average SASA"), the conformation with the smallest SASA ("Min SASA"), or the conformation corresponding to the most probable SASA value ("Most probable SASA"). The free energies generated for each conformation were compared to the free energies obtained from umbrella sampling using molecular dynamics (Figure 3 of the main text). The results indicate that the "Most probable SASA" approach yields reasonable agreement across the entire data set.

Table S1. Comparison of COSMOmic free energies computed for different molecular conformations. The absolute error is calculated relative to the values determined using umbrella sampling simulations using molecular dynamics. All values are kJ/mol.

	**C4-AHL**	**Abs. Error**	**3-oxo-C12-AHL**	**Abs. Error**
	ΔG_{par}			
Max SASA	*-0.76 ± 0.79*	*6.93*	*-17.16 ± 0.70*	*2.88*
Average SASA	*1.71 ± 0.81*	*9.41*	*-9.84 ± 0.55*	*10.19*
Min SASA	*-0.98 ± 0.69*	*6.72*	*-11.83 ± 0.60*	*8.20*
Most probable SASA	*-0.94 ± 0.40*	*6.76*	*-11.90 ± 1.12*	*8.14*
	ΔG_{trans}			
Max SASA	*38.08 ± 0.62*	*8.38*	*23.77 ± 0.77*	*9.81*
Average SASA	*36.05 ± 0.56*	*6.35*	*26.52 ± 2.01*	*7.06*
Min SASA	*33.13 ± 0.35*	*3.43*	*36.92 ± 0.74*	*3.34*
Most probable SASA	*30.74 ± 0.68*	*1.04*	*28.47 ± 1.19*	*5.11*

Figure S2 shows potentials of mean force (PMFs) for the partitioning and translocation of 3-oxo-C12-AHL as a function of the distance, *z*, normal to the DOPC membrane computed using COSMOmic. Three PMFs were generated using different numbers of molecular orientations ($N_{orientation}$ = 12, 162 and 362). All three PMFs show comparable results. We chose to use 162 orientations for all results presented in the main text.

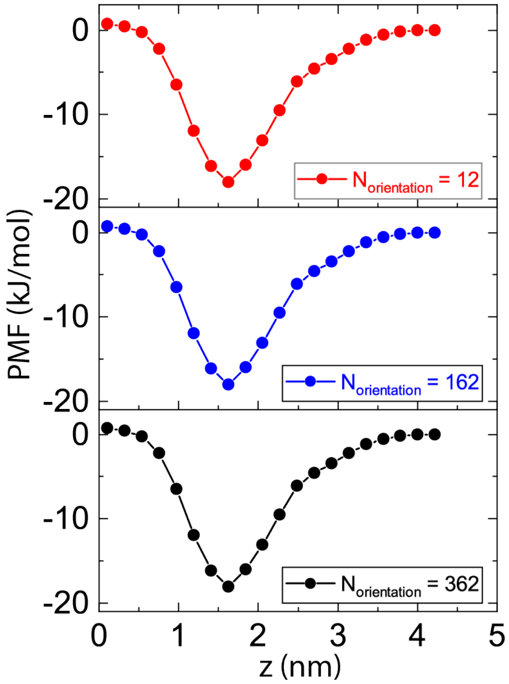

Figure S2. Potentials of mean force (PMFs) computed using COSMOmic for 3-oxo-C12-AHL as a function of the distance, *z*, along the DOPC membrane normal with different numbers of molecular orientations.

Figure S3 shows potentials of mean force (PMFs) for the partitioning and translocation of 3-oxo-C12-AHL as a function of the distance, *z*, normal to the DOPC membrane computed using COSMOmic. Three PMFs were generated using different numbers of slices (N_{slice} = 10, 20 and 30). The PMFs for N_{slice} = 20 and N_{slice} = 30 were nearly identical, whereas the PMF for N_{slice} = 10 differed from the other two. We selected N_{slice} = 20 for all results presented in the main text; additional slices yield no change in accuracy while requiring additional computational time. s

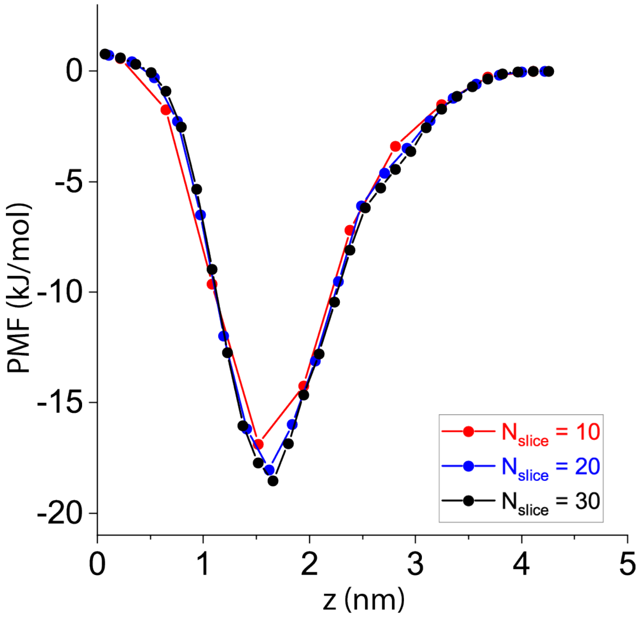

Figure S3. Potentials of mean force (PMFs) computed using COSMOmic for 3-oxo-C12-AHL as a function of the distance, *z*, along the DOPC membrane normal with different numbers of membrane slices.

Additional umbrella sampling results

Figure S4 shows evidence for the convergence of the PMFs for C4-AHL, C8-AHL, C12-AHL and 3-oxo-C12-AHL, each plotted as a function of the distance, *z*, along the DOPC membrane normal. PMFs are computed using umbrella sampling with the weighted histogram analysis method (WHAM). Lines of different colors indicate the total sampling time used in each simulation window to compute the PMF, excluding an initial 8 ns of simulation time that were used to account for equilibration. In each case, the PMFs converge by 20 ns of simulation time.

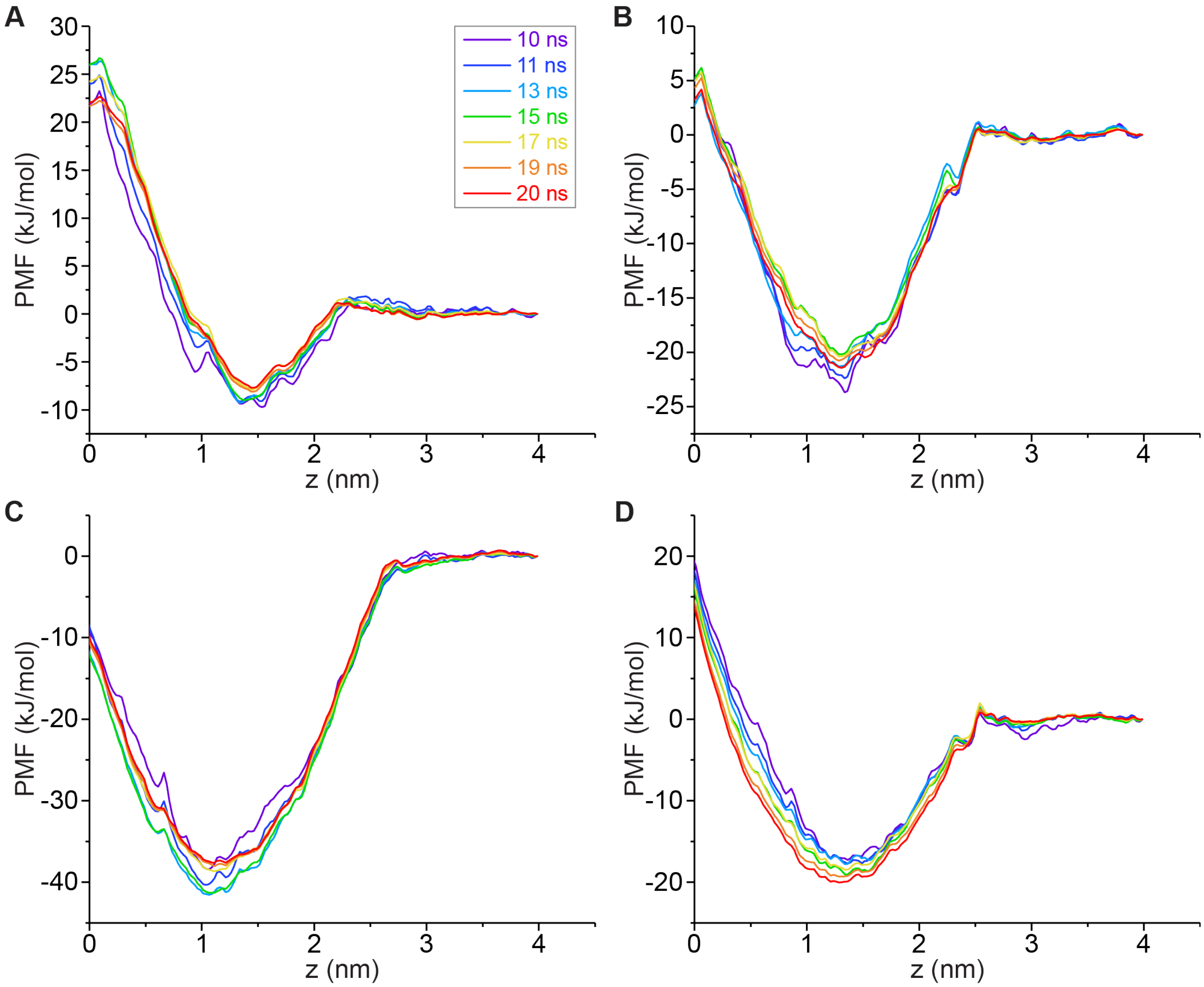

Figure S4. Convergence of the potentials of mean force (PMFs) computed using umbrella sampling as a function of the distance, *z*, along the DOPC membrane normal. PMFs are shown for **A)** C4-AHL, **B)** C8-AHL, **C)** C12-AHL and **D)** 3-oxo-C12-AHL.

Table S2 compares the partition (ΔG_{par}) and translocation (ΔG_{trans}) free energies calculated using umbrella sampling to the same values computed using COSMOmic protocol for C4-AHL, C8-AHL, C12-AHL and 3-oxo-C12-AHL. ΔG_{trans} values are shown both directly from COSMOmic and after using the extrapolation approach described in the main text is shown for COSMOmic protocol, with the latter approach leading to better agreement with the umbrella sampling results.

Table S2. Comparison of partition (ΔG_{par}) and translocation (ΔG_{trans}) free energies calculated using umbrella sampling and COSMOmic for C4-AHL, C8-AHL, C12-AHL and 3-oxo-C12-AHL. All values are kJ/mol.

	**Umbrella Sampling**		**COSMOmic**		
	ΔG_{par}	ΔG_{trans}	ΔG_{par}	ΔG_{trans} (raw)	ΔG_{trans} (extrapolated)
C4-AHL	*-6.36 ± 1.22*	*28.63 ± 1.23*	*-0.94 ± 0.40*	*19.46 ± 0.57*	*30.74 ± 0.68*
C8-AHL	*-21.2 ± 0.22*	*25.72 ± 0.96*	*-8.91 ± 0.41*	*16.59 ± 0.93*	*27.99 ± 1.05*
C12-AHL	*-36.79 ± 0.84*	*29.14 ± 1.72*	*-17.57 ± 1.22*	*14.46 ± 1.50*	*26.06 ± 1.56*
3-oxo-C12AHL	*-23.16 ± 3.13*	*35.18 ± 3.37*	*-11.90 ± 1.12*	*15.87 ± 1.75*	*28.47 ± 1.19*

Correlation between COSMOmic translocation free energy methods

Figure S5 shows QSM translocation free energies (ΔG_{trans}) calculated either as the free energy difference between z = 0 and the plateau value of the PMF, which is labeled as ΔG_{trans} (z = 0), or by extrapolating a line fit to the third, fourth and fifth data points in the COSMOmic PMF to z = 0, which is labeled as ΔG_{trans} (extrapolation). Both approaches are linearly correlated with r^2 = 0.98, indicating that the rank-ordered trend of translocation free energies is unaffected. The linear extrapolation procedure more closely represents the values from umbrella sampling (Table S2).

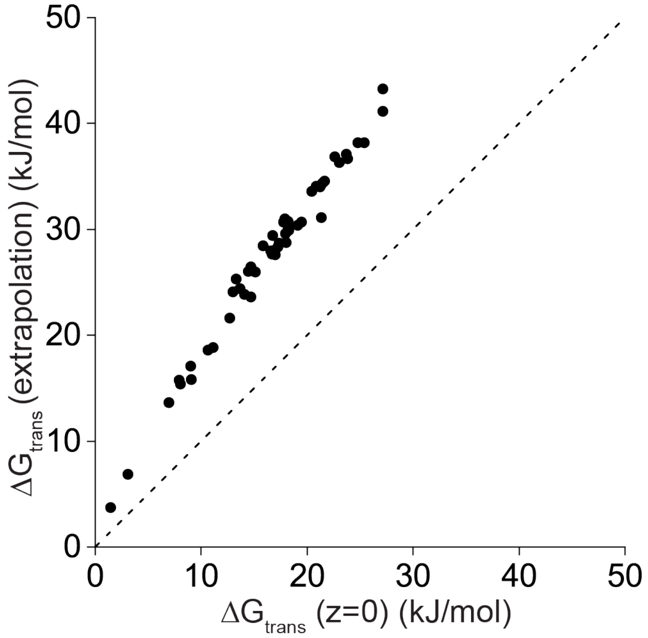

Figure S5. Comparison of translocation free energies (ΔG_{trans}) computed using COSMOmic determined either directly from the PMF (x-axis) or by linear extrapolation (y-axis). The dashed line indicates $y = x$.

Tabulated experimental data

Table S3 lists experimental measurements of LasR activation (EC_{50}) and inhibition (IC_{50}) for various QSMs and corresponding values of ΔG_{par} and ΔG_{trans}. These data are used in Figure 8 of the main text. Experimental data are taken from past literature references as listed [1-9]. Inhibition experiments were conducted in either *E. coli* or *P. aeruginosa* whereas activation experiments were performed in *E. coli*.

Table S3. Experimental measurements of LasR activation (EC_{50}) and inhibition (IC_{50}) and corresponding values ΔG_{par} and ΔG_{trans} for various quorum sensing modulators.

**Compound**	**ΔG_{par} (kJ/mol)**	**ΔG_{trans} (kJ/mol)**	**EC_{50} (μM) (*E. coli.*)**	**IC_{50} (μM) (*E. coli.*)**	**IC_{50} (*P. aeruginosa*)**	**Ref.**
A3 (OHL)	*-8.91*	*27.99*		*1.52*		3, 5
A4 (DHL)	*-11.25*	*24.13*		*0.25*	*1*	5
B11	*-5.89*	*25.97*		*1.75*		5
B7	*-7.33*	*29.94*	*8.4*	*0.36*	*12*	2
C14	*-5.82*	*37.11*		*0.61*		5
C6	*-5.65*	*34.43*		*3.97*		5
C8	*-6.86*	*28.70*		*4.06*		5
D15	*-7.73*	*23.89*	*6.3*	*4.67*		5
F6	*-7.90*	*18.63*		*0.79*		8
OHHL	*-1.47*	*30.41*		*10.4*	*40*	2
S7	*-8.03*	*24.40*		*10*		7
B2	*-2.70*	*43.27*	*0.54*			5
D18	*-20.30*	*31.16*	*0.47*			5
E8	*-9.53*	*30.80*	*3.4*			4
F1	*-19.40*	*21.66*	*1.5*			8
F11	*-9.35*	*28.77*	*4.6*			8
F15	*-17.09*	*6.91*	*0.16*			9
F3	*-3.75*	*36.71*	*0.13*			8
F4	*-22.08*	*15.44*	*1.9*			8
E35	*-7.21*	*30.09*			*4.35*	1
E5	*-3.28*	*41.15*			*12*	1

References

1. Moore JD, Gerdt JP, Eibergen NR, Blackwell HE. Active Efflux Influences the Potency of Quorum Sensing Inhibitors in Pseudomonas aeruginosa. Chembiochem. 2014;15(5):634-.
2. Moore JD, Rossi FM, Welsh MA, Nyffeler KE, Blackwell HE. A Comparative Analysis of Synthetic Quorum Sensing Modulators in Pseudomonas aeruginosa: New Insights into Mechanism, Active Efflux Susceptibility, Phenotypic Response, and Next-Generation Ligand Design. J Am Chem Soc. 2015;137(46):14626-39.
3. Gerdt JP, Wittenwyler DM, Combs JB, Boursier ME, Brummond JW, Xu H, et al. Chemical Interrogation of LuxR-type Quorum Sensing Receptors Reveals New Insights into Receptor Selectivity and the Potential for Interspecies Bacterial Signaling. Acs Chem Biol. 2017;12(9):2457-64.
4. Geske GD, Mattmann ME, Blackwell HE. Evaluation of a focused library of N-aryl L-homoserine lactones reveals a new set of potent quorum sensing modulators. Bioorg Med Chem Lett. 2008;18(22):5978-81.
5. Geske GD, O'Neill JC, Miller DM, Mattmann ME, Blackwell HE. Modulation of bacterial quorum sensing with synthetic ligands: Systematic evaluation of N-acylated homoserine lactones in multiple species and new insights into their mechanisms of action. J Am Chem Soc. 2007;129(44):13613-25.
6. Gerdt JP, McInnis CE, Schell TL, Rossi FM, Blackwell HE. Mutational Analysis of the Quorum-Sensing Receptor LasR Reveals Interactions that Govern Activation and Inhibition by Nonlactone Ligands. Chem Biol. 2014;21(10):1361-9.
7. Mattmann ME, Shipway PM, Heth NJ, Blackwell HE. Potent and Selective Synthetic Modulators of a Quorum Sensing Repressor in Pseudomonas aeruginosa Identified from Second-Generation Libraries of N-Acylated L-Homoserine Lactones. Chembiochem. 2011;12(6):942-9.
8. McInnis CE, Blackwell HE. Thiolactone modulators of quorum sensing revealed through library design and screening. Bioorgan Med Chem. 2011;19(16):4820-8.
9. Boursier ME, Manson DE, Combs JB, Blackwell HE. A comparative study of non-native N-acyl L-homoserine lactone analogs in two Pseudomonas aeruginosa quorum sensing receptors that share a common native ligand yet inversely regulate virulence. Bioorgan Med Chem. 2018;26(19):5336-42.
